# Mindfulness-Based Resilience Training in the Workplace: Pilot Study of the Internet-Based Resilience@Work (RAW) Mindfulness Program

**DOI:** 10.2196/10326

**Published:** 2018-09-11

**Authors:** Sadhbh Joyce, Fiona Shand, Richard A Bryant, Tara J Lal, Samuel B Harvey

**Affiliations:** 1 School of Psychiatry Faculty of Medicine University of New South Wales Randwick Australia; 2 Black Dog Institute Randwick Australia; 3 School of Psychology Faculty of Science University of New South Wales Randwick Australia; 4 Fire and Rescue New South Wales Alexandria Australia

**Keywords:** resilience training, workplace mental health, occupational health, wellbeing, online intervention, employee resilience, health and safety, psychological health

## Abstract

**Background:**

The impact of mental illness on society is far reaching and has been identified as the leading cause of sickness absence and work disability in most developed countries. By developing evidence-based solutions that are practical, affordable, and accessible, there is potential to deliver substantial economic benefits while improving the lives of individual workers. Academic and industry groups are now responding to this public health issue. A key focus is on developing practical solutions that enhance the mental health and psychological resilience of workers. A growing body of research suggests resilience training may play a pivotal role in the realm of public health and prevention, particularly with regards to protecting the long-term well-being of workers.

**Objective:**

Our aim is to examine whether a mindfulness-based resilience-training program delivered via the internet is feasible and engaging to a group of high-risk workers. Additionally, we aim to measure the effect of the Resilience@Work Resilience@Work Mindfulness program on measures of resilience and related skills.

**Methods:**

The current pilot study recruited 29 full-time firefighters. Participants were enrolled in the 6-session internet-based resilience-training program and were administered questionnaires prior to training and directly after the program ended. Measurements examined program feasibility, psychological resilience, experiential avoidance, and thought entanglement.

**Results:**

Participants reported greater levels of resilience after Resilience@Work training compared to baseline, with a mean increase in their overall resilience score of 1.5 (95% CI -0.25 to 3.18, *t*_14_=1.84, *P*=.09). Compared to baseline, participants also reported lower levels of psychological inflexibility and experiential avoidance following training, with a mean decrease of -1.8 (95% CI -3.78 to 0.20, *t*_13_=-1.94, *P*=.07). With regards to cognitive fusion (thought entanglement), paired-samples *t* tests revealed a trend towards reduction in mean scores post training (*P*=.12).

**Conclusions:**

This pilot study of the Resilience@Work program suggests that a mindfulness-based resilience program delivered via the Internet is feasible in a high-risk workplace setting. In addition, the firefighters using the program showed a trend toward increased resilience and psychological flexibility. Despite a number of limitations, the results of this pilot study provide some valuable insights into what form of resilience training may be viable in occupational settings particularly among those considered high risk, such as emergency workers. To the best of our knowledge, this is the first time a mindfulness-based resilience-training program delivered wholly via the internet has been tested in the workplace.

## Introduction

Improving workplace mental health is an opportunity of immense scale and profound importance [1-3]. By developing evidence-based solutions that are practical, affordable, and accessible, there is potential to deliver substantial economic benefits, while improving the lives of individual workers [4,5]. The impact of mental illness on society is far reaching and has been identified as the leading cause of sickness absence and work disability in most developed countries [6-11]. Poor mental health also produces large productivity losses due to absenteeism as well as presenteeism, with affected workers attending work, yet performing at a diminished capacity [12,13]. As a result, common mental health conditions such as depression and anxiety have a significant and direct impact on the overall economic welfare of a nation [14,15]. However, the impact of mental illness in the working population goes well beyond macroeconomics. Once an individual worker develops a mental health condition, they often suffer personal financial losses, career disruption, and reduced well-being.

Academic and industry groups are now responding to this public health issue. A key focus is on developing practical solutions that enhance the mental health and psychological resilience of workers [16]. There is no simple universal solution to workplace mental health. Best practice frameworks highlight the importance of a multifaceted approach that addresses individual, team, and organizational level factors. These factors include work design, organizational culture, good management, promoting and facilitating early help-seeking and early intervention, as well as supporting return-to-work programs and recovery [16,17]. These frameworks also make specific reference to the importance of employee resilience training. This type of individual training can form part of broader programs of workplace health promotion [18].

Indeed, a growing body of research suggests resilience training may play a pivotal role in the realm of public health and prevention, particularly with regards to protecting the long-term well-being of workers [17,19,20]. While definitions of resilience are diverse and plentiful, there is growing consensus that resilience is a malleable construct, wherein an individual’s ability to adapt effectively during challenging circumstances can be enhanced over time. Leading researchers in the field, along with the American Psychological Society, describe resilience as a process of “bouncing back” from difficult experiences and “adapting well in the face of adversity, trauma, tragedy, threats or significant sources of stress” [21,22].

In terms of enhancing resilience, numerous studies have described positive outcomes from various types of resilience training programs among groups including medical specialists, youth workers, nurses, factory workers, and public servants [23-28]. In addition, research among emergency workers (ie, firefighters, police, paramedics) and military personnel highlights the benefits of resilience training among individuals who frequently experience high-stress situations as an inherent aspect of their work [29-31]. Conversely, a number of larger trials with US Army Personnel and more recently with London Ambulance in the United Kingdom reported limited improvements following resilience training [32,33]. Establishing what types of resilience training programs are beneficial to high-risk groups such as emergency workers is particularly important for several reasons. First, these workers play an essential role in delivering and maintaining critical services in our communities. Second, given the nature of their work, emergency workers are at greater risk of developing common mental health conditions such as depression, anxiety, and alcohol misuse as well as posttraumatic stress disorder (PTSD) [34-37]. Finally, resilience programs that are evaluated and found to be useful among emergency service personnel may provide valuable insight on how to best support the mental health of workers in other high-stress occupations (eg, health care, journalism).

Despite the growing body of research supporting resilience training, considerable measurement variation exists in terms of how researchers evaluate the effectiveness of these programs. For example, some researchers specifically focus on changes observed on reliable and validated measures of psychological resilience following times of intense stress and adversity. Windle et al [38] offer a review of resilience measures. Other researchers have primarily examined the overall impact of resilience training on measures of general well-being and mental health symptomology. While research continues to highlight a positive relationship between resilience and psychological well-being, the latter approach may provide limited insight into whether a resilience intervention can truly facilitate change in an individual’s overall ability to bounce back from adversity. A program may improve mental health symptoms, yet not enhance a person’s overall psychological resilience or vice versa [39,40]. The use of reliable and validated measures of psychological resilience is central to examining the efficacy of any intervention aimed at enhancing psychological resilience [38], particularly in groups where people identify as “mentally healthy.”

Resilience training programs can differ considerably in terms of content, delivery, and length. In their systematic review of resilience interventions, Leppin et al unsurprisingly concluded “no single accepted theoretical framework or consensus statement exists to guide the development or application of these programs” [19]. This may explain why resilience researchers are now drawing on evidence-based therapies such as Acceptance and Commitment Therapy (ACT), Cognitive Behavioral Therapy, Mindfulness-Based Cognitive Therapy, and Mindfulness-Based Stress Reduction (used in the treatment of common mental health conditions) to inform program development [23,24,27,41-45]. These resilience programs tend to include a combination of cognitive strategies, mindfulness training, psycho-educational material, and goal setting. They typically focus on enhancing a person’s capacity to manage stressful situations and adverse circumstances more effectively and with greater emotional insight. These skills and strategies require time to practice and gain proficiency. As such, the majority of resilience studies to date describe interventions involving multiple face-to-face training sessions [19,20]. This is a particular challenge for many employers, where taking workers away from the workplace to attend training creates considerable disruption to business and critical services. In addition, the associated costs for replacement staff during this time can be significant. The expense inherent in face-to-face training can pose a hindrance, as can the availability of trainers and programs in remote areas. Moreover, stigma associated with mental health remains prevalent and may prevent a subset of workers from choosing to engage openly in group-training sessions that focus on psychological topics [46]. A universal approach where all employees complete the training may go some way towards reducing this stigma [47].

**Figure 1:**
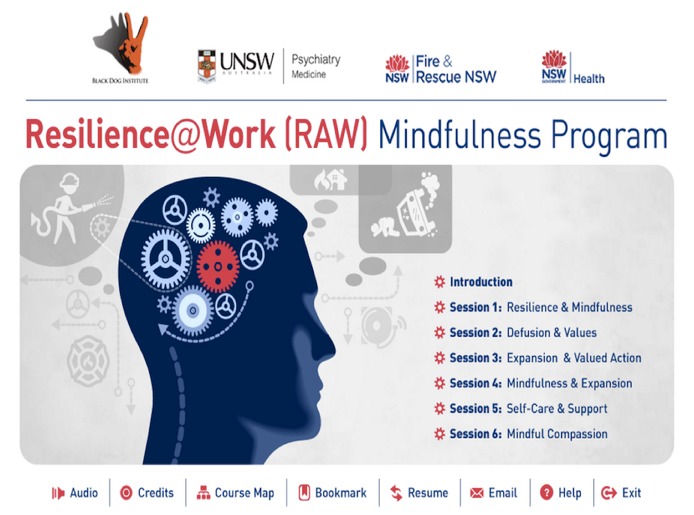
Resilience@Work Mindfulness Program homepage.

To address these barriers, we developed an interactive e-learning program called The Resilience@Work (RAW) Mindfulness Program. This self-paced intervention aims to enhance psychological resilience among workers. It consists of 6 internet-based training sessions, each taking about 20-25 minutes to complete on a tablet or computer (see Figure 1).

The RAW program involves mindfulness training, psycho-education, and a range of skills and strategies drawn from evidence-based therapies including ACT, Mindfulness-Based Stress Reduction, and Compassion-Focused Therapy. A large body of literature highlights the positive benefits of mindfulness practice on mental health outcomes [48-53] while a growing number of studies also describe the positive impact of mindfulness training on psychological resilience [23,24,41,54].

The RAW program also teaches a number of core cognitive strategies, which may further enhance a learner’s ability to manage stress and cope with adverse circumstances more effectively. These core strategies, drawn from ACT, aim to enhance psychological flexibility by applying mindfulness, acceptance-based emotion regulation strategies, and cognitive skills, while also emphasizing behavioral change that reflects personal values. Psychological flexibility is “the ability to be in the present moment with full awareness and openness to our experience, and to take action guided by our values” [55]. Psychological flexibility is associated with lower levels of depression, anxiety, and distress in clinical and nonclinical populations [56-58]. More recently, it has been found to protect against depression and PTSD among returned service personnel [59].

**Table 1:** Overview of skills and topics covered in the Resilience@Work Mindfulness Program.

Session	Resilience topic and skills focus	Mindfulness tracks
1	Introduction to mindfulness, resilience and psychological well-being	Drop Anchor Take 10 Leaves on a Stream
2	Mindfulness skills, Understanding your reactive mind versus wise mind, Recognizing unhelpful mind chatter and managing uncomfortable and unhelpful thoughts (cognitive defusion); Recognizing your values exercise	Mindful Breathing Defusion Technique; Notice it, Name it, Let it Go (I’m having the thought that…) Defusion Technique 2: Thank you Mind
3	Revision of cognitive defusion, Introduction to mindfulness with emotions, The reactive mind and avoidance, Understanding how values are linked to emotions; Valued action check	Creating Space (mindfulness with emotions) Mindful Body Scan The Golden Room
4	The problem with avoidance, Recognizing avoidance strategies versus adaptive strategies	Creating Space A Mindful Break (mindfulness with words) Surfing Waves
5	Self-care and support, The compassion myth, barriers to accessing compassion, compassion fatigue, self-compassion actions & resilience; Identifying mindful support (compassionate, nonjudgmental and mindful); Valued action check	A Kind and Gentle Hand (loving-kindness practice) A Safe Place (compassion-focused mindfulness) A Bird’s Eye View
6	Compassion-focused mindfulness; Gratitude practice, optimism and resilience, identify and celebrate the milestones; Creating a personalized action plan to practice skills	Breathing in the Present Moment A Golden Moment exercise Being Kind to your old wounds

Table 1 provides an overview of the resilience topics, core strategies, and mindfulness skills covered in each session. Several reviews and meta-analyses have found medium to large effect sizes for ACT-based interventions across a range of clinical and nonclinical settings including anxiety, depression, substance abuse, worksite stress, and burnout [60-64]. Moreover, a number of studies have found that ACT can improve mental health in the workplace [64,65], highlighting its potential as an intervention that may promote psychological resilience in occupational settings.

A recent review and meta-analysis found that digital mental health interventions in the workplace can improve psychological well-being and work effectiveness among employees [66]. Despite the apparent advantages of online resilience training, there has been very limited research examining the acceptability and efficacy of this approach. A few trials have examined either a blended approach (ie, programs that combine internet-based and face-to-face resilience training) [24,41] or an online approach with an emphasis on stress reduction and/or enhancing resilience-related factors [67,68]. As with the main resilience literature to date, these studies vary greatly in their approach to measuring program efficacy and thus limited conclusions can be drawn. In addition, while the research evidence for online mindfulness interventions continues to grow [48,69], to date there have been no published trials examining the efficacy of a mindfulness-based resilience training program delivered solely online.

The primary aim of our pilot study is to examine whether a mindfulness-based resilience-training program delivered via the internet is feasible and engaging to a high-risk group of workers, that is, firefighters. A secondary aim is to capture changes in measures of resilience and psychological skills among firefighters undertaking the training program. To the best of our knowledge, this is the first pilot study of a self-paced mindfulness-based resilience training program delivered completely in an online format.

## Methods

### Resilience@Work Mindfulness Program

**Figure 2:**
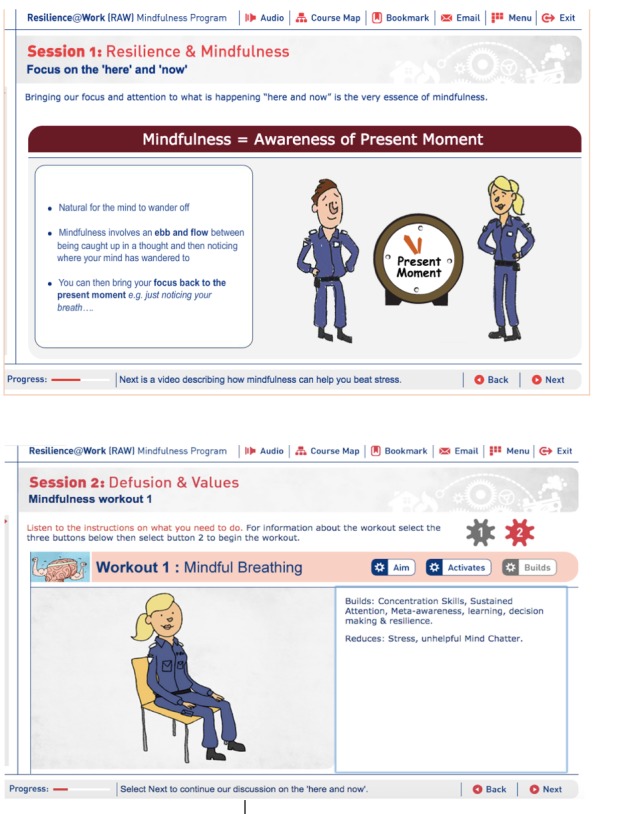
Screenshots of training material from the Resilience@Work Mindfulness Program

The RAW program is a mindfulness-based intervention, which also draws on ACT and has significant emphasis on self-compassion and acceptance skills. The intervention involves completing 6 internet-based training sessions. Each session takes about 20-25 minutes to complete on a tablet or computer. It was anticipated that an engaging and interactive program would help address the issue of adherence; a challenge that employers frequently encounter when offering resilience training and support to their workers. Rather than having to read through lengthy paragraphs on a website, the RAW program engages workers in the process of learning by utilizing a combination of interactive exercises, audio, and animation (see Figure 2).

Participants were able to download mindfulness tracks to their own device for continued practice. Participants also had the opportunity to sign up for text-message reminders and/or reminder emails. A podcast accompanied each RAW session with additional mindfulness tracks to encourage skills development. Podcasts were not a mandatory part of the training but were available via a website for those participants who chose to use them.

To ensure program engagement, workers from target industries were invited to provide detailed insight into the specific work-related challenges they encountered on a regular basis. Examples were provided by workers to the research team via email, phone, and in person during a workplace well-being seminar. This information was collated and incorporated throughout the RAW program as “real-world” examples when introducing new resilience strategies and techniques.

Each session teaches a new strategy to cultivate psychological resilience and involves a combination of psycho-education and mindfulness training. The program also interweaves simple quotes and messages from the eastern philosophies of Buddhism and Yogic teaching traditions from which mindfulness has its origins [70-74].

### Participants

Participants for this pilot study were drawn from Fire and Rescue New South Wales (FRNSW) in Australia. FRNSW is the seventh largest urban fire service in the world and responds to firefighting, rescue, and hazardous material emergencies in Sydney, Australia, and surrounding regional areas. Given the nature of their work, employees are known to have elevated risk of depression, anxiety, and PTSD [36].

Potential participants were informed about the study during a standard well-being talk facilitated by members of the FRNSW Peer Support Team. Firefighters were provided with a participant information sheet and consent form to read and review along with the study questionnaire. Participation was voluntary. Firefighters who opted to participate in the study signed the consent form and provided a valid email address in order to register into the training program. Prepaid envelopes were provided to mail consent forms and completed questionnaires to the research team. Overall, 29 firefighters were recruited (see Table 2). Any potential participants who were currently engaged in any regular individual psychological therapy sessions with a psychologist and/or psychiatrist were excluded from this study. Ethics approval was obtained via the Human Research Ethics Committee at the University of New South Wales, Australia.

### Measures

The current pilot study sought to (1) examine the initial feasibility of the RAW Mindfulness Program in a workplace setting and (2) determine whether it would lead to measurable changes in resilience and key process variables, specifically cognitive fusion and experiential avoidance.

#### Measure of Feasibility

Engagement and feasibility of the RAW Mindfulness Program were recorded by storing the total number of sessions completed by each participant and the number of training hours completed.

#### Measure of Resilience

Psychological resilience was measured using the validated short form 10-item version of the Connor-Davidson Resilience Scale (CD-RISC 10) [75]. Participants respond to each item on a 5-point scale, ranging from 0 (not true at all) to 4 (true nearly all of the time). The total score ranges from 0-40 with a higher score indicative of higher psychological resilience. Previous studies have found the CD-RISC 10 to be a reliable and valid measure with Cronbach alpha ranging from .81-.88 [76,77] and test-retest reliability of 0.9 at 6 weeks [77,78].

#### Measure of Process

The RAW Mindfulness Program was designed to utilize a variety of mindfulness and ACT techniques, the most prominent of which were cognitive defusion and psychological flexibility. In order to measure the impact of the intervention program on these processes, the Cognitive Fusion Questionnaire (CFQ) and the Acceptance and Action Questionnaire version 2 (AAQ-II) were administered to participants.

##### Cognitive Fusion Questionnaire

The CFQ is a measure of cognitive fusion and defusion, a core component of the ACT model [79]. The CFQ contains 7 items rated on a 7-point scale from 1 (never true) to 7 (always true) with a total score range of 7-49. A higher score reflects greater cognitive fusion and thought entanglement. A sample item is “I get so caught up in my thoughts that I am unable to do the things that I most want to do.” Previous studies have found the CFQ to be a reliable and valid measure with Cronbach alpha ranging from .89-.93 [79,80].

**Table 2:** Demographics of participants in Resilience@Work pilot study (N=29).

Characteristics		Value
Age, mean (SD); range		43.7 (8.7) 24-59
**Sex, n (%)**		
	Male	28 (97)
	Female	1 (3)
**Highest education, n (%)**		
	High school	8 (27.6)
	Technical and Further Education (TAFE)	15 (51.7)
	Graduate degree	5 (17.2)
	Postgraduate degree	1 (3.4)
**Years with Fire and Rescue New South Wales** **, n (%)**		
	1-5	3 (10.7)
	6-10	4 (14.3)
	11-15	5 (17.9)
	16-20	3 (10.7)
	20+	13 (46.4)

##### Acceptance and Action Questionnaire-II

The AAQ-II is a 7-item self-reported measure of experiential avoidance and psychological inflexibility. Participants rate each question on a 7-point Likert scale from 1 (never true) to 7 (always true) with a total score range of 7-49. A higher score reflects greater avoidance behavior and less psychological flexibility. Previous research has found the AAQ-II to be a reliable and valid measure with a Cronbach alpha of .84 and test-retest reliability of 0.81 at 3-month follow-up [56].

#### Data Analysis Plan

Analyses were conducted using SPSS statistical analysis program. Prior to analysis, frequency distributions and plots for each of the outcome and process variables were examined for unusual data points and to ensure the assumption of normality was not violated, using the Shapiro-Wilk’s test. Paired-samples *t* tests were used to determine any differences between each measure at baseline and immediately after the intervention. The main measure of the efficacy of the intervention was the level of psychological resilience as measured by the CD-RISC 10. We proposed that an effect size of 0.5 would be considered a meaningful and clinically important effect. Based on such figures, we aimed to recruit at least 26 participants to this pilot study, which would achieve 0.8 power of detecting an effect size of 0.5 in terms of the CD-RISC 10 with an alpha of 0.1 (two-sided). This approach is similar to other pilot studies of this kind [81]. The total number of modules completed by each participant was also recorded to examine program engagement. In addition, univariate analysis using chi-square tests and Student *t* tests were used to examine which baseline measures predicted completion of at least 50% of the RAW program. Baseline factors considered were age, gender, level of education, years working as a firefighter, and baseline resilience.

## Results

### Overview

A total of 29 firefighters were recruited for the pilot study. Of the participants, 72% (21/29) had completed some form of post-high school education and the majority (16/29, 55%) had been employed by FRNSW for more than 15 years. In line with most first responder agencies, the vast majority of participants were male. Baseline resilience scores on the CD-RISC 10 were similar to normative data from first responders [69].

### Program Engagement

Table 3 outlines the number of RAW program sessions completed by participants. The majority of participants (16/29, 55%) completed more than half the program (mean number of sessions completed was 3.6 out of a possible 6, SD 2.2) equating to 60-75 minutes of training. Eleven participants (11/29, 38%) completed all 6 sessions (a total of at least 2 hours training).

Analysis examining for baseline predictors of completion found no evidence that age, gender, level of education, years working as a firefighter, or baseline resilience were able to predict which participants were more likely to complete at least half of the RAW program (*P*>.05 for all).

### Resilience, Cognitive Fusion, and Psychological Inflexibility/Experiential Avoidance

Participants reported greater levels of resilience after RAW training compared to baseline, with a mean increase in their CD-RISC 10 score of 1.5 (95% CI -0.25 to 3.18, *t*_14_=1.84, *P*=.09), equating to a moderate effect size of 0.5. Table 4 displays the baseline and post-training measurements of resilience and measures of process.

**Table 3:** The number of Resilience@Work sessions completed by pilot study participants.

Minimum number of sessions completed	n (%)
1	29 (100)
2+	21 (72)
3+	16 (55)
4+	14 (48)
5+	14 (48)
6	11 (38)

**Table 4:** Baseline and post-training scores for measures of resilience and process variables.

Measure	Baseline, mean (SD)	Post Resilience@Work training, mean (SD)	*P* value
Resilience, CD-RISC 10^a^ (n=15)	26.0 (5.5)	27.5 (4.9)	.09
Cognitive fusion, CFQ^b^ (n=13)	20.7 (8.9)	18.4 (7.5)	.12
Psychological inflexibility, AAQ-II^c^ (n=14)	18.5 (6.7)	16.7 (5.7)	.07

^a^CD-RISC 10: 10-item version of the Connor-Davidson Resilience Scale.

^b^CFQ: Cognitive Fusion Questionnaire.

^c^AAQ-II: Acceptance and Action Questionnaire version 2.

Compared to baseline, participants reported lower levels of psychological inflexibility and experiential avoidance following training, with a mean decrease of -1.8 (95% CI -3.78 to 0.20, *t*_13_=-1.94, *P*=.07). With regards to cognitive fusion (thought entanglement), paired-samples *t* test revealed a trend towards reduction in mean scores post training (*P*=.12).

## Discussion

### Principal Findings

This pilot study of the RAW Mindfulness Program suggests that an internet-based resilience-training program is feasible in a workplace setting. In addition, those using the RAW program showed a trend toward increased resilience and psychological flexibility. To the best of our knowledge, this is the first time a wholly online mindfulness-based resilience-training program and its feasibility have been tested in the workplace.

While it is difficult to directly compare effect sizes from pre-post studies compared to control trials, it is worth noting that the moderate effect sizes demonstrated in this pilot study are similar to those described in a recent meta-analysis examining the effectiveness of online mindfulness interventions aimed at reducing stress [48]. In addition, the observed trends in both of the predicted process factors, cognitive fusion (thought entanglement), and psychological inflexibility/experiential avoidance, suggest the desired skills and techniques can be taught via an internet-based format.

### Limitations

There were some important limitations to this pilot study, most notably the lack of a control group, the small sample size, and the absence of longer-term follow-up. The use of self-reported measures of resilience and process measures is also a limitation, although all scales used were well validated and the resilience measure chosen is known to be associated with a range of mental health outcomes among working populations [82]. Recruitment was facilitated by peer supporters and occurred while a proportion of firefighters were either responding to emergency calls or off duty. It is therefore unknown what proportion of firefighters were informed of the program and subsequently signed up for resilience training. Thus, limited insight was gained into overall acceptability of the program. It is important to note that our sample of emergency workers was a uniformed, male-dominated, high-risk group. Therefore, it remains unclear as to whether this form of resilience training is feasible among gender-balanced, low-risk workforces.

While most participants completed half of the program, there was a notable drop in completion after the second session. This may be due to a new cognitive skill being taught in this session that focused on how to manage difficult and uncomfortable thoughts. This may have been particularly confronting or challenging for some learners. Dropout analysis found that level of baseline resilience, age, gender, education level, and years on the job did not predict who would go on to complete more than 50% of the program. It is worth noting that this analysis is hindered by an overall lack of power and that other factors such as intrinsic motivation may have influenced completion rates. That said, most participants completed at least half of the RAW program and of these most went on to complete the entire program (ie, all 6 sessions).

### Conclusion

Despite these limitations, the results of this pilot study provide some valuable insights into what form of resilience training may be viable in occupational settings. More specifically, it suggests that internet-based resilience training is a feasible approach in workplaces, particularly among those considered high risk, such as first responders, and those with specific inherent challenges for training, such as shift work, frequent travel on the road, and limited access to face-to-face training.

In spite of these promising results, the effectiveness of the RAW Mindfulness Program needs to be tested via a larger randomized controlled trial, ideally with both short-term and longer-term follow up. Additional secondary outcome measures, such as levels of psychological symptoms, perceived stress, and well-being are also needed to establish whether programs such as the RAW program can create meaningful changes beyond short-term gains in self-reported resilience.

## Abbreviations

AAQ-II: Acceptance and Action Questionnaire version 2
ACT: acceptance and commitment therapy
CD-RISC 10: 10-item version of the Connor-Davidson Resilience Scale
CFQ: Cognitive Fusion Questionnaire
FRNSW: Fire and Rescue New South Wales
NSW: New South Wales
PTSD: posttraumatic stress disorder
RAW: Resilience@Work
